# A Comparison of Local Path Planning Techniques of Autonomous Surface Vehicles for Monitoring Applications: The Ypacarai Lake Case-study

**DOI:** 10.3390/s20051488

**Published:** 2020-03-09

**Authors:** Federico Peralta, Mario Arzamendia, Derlis Gregor, Daniel G. Reina, Sergio Toral

**Affiliations:** 1 Facultad de Ingeniería, Universidad Nacional de Asunción, 2160 San Lorenzo, Paraguaymarzamendia@ing.una.py (M.A.); dgregor@ing.una.py (D.G.); 2 Universidad de Sevilla, 41004 Sevilla, Espana

**Keywords:** autonomous surface vehicle, local path planning, monitoring applications, motion planning, Ypacarai lake

## Abstract

Local path planning is important in the development of autonomous vehicles since it allows a vehicle to adapt their movements to dynamic environments, for instance, when obstacles are detected. This work presents an evaluation of the performance of different local path planning techniques for an Autonomous Surface Vehicle, using a custom-made simulator based on the open-source Robotarium framework. The conducted simulations allow to verify, compare and visualize the solutions of the different techniques. The selected techniques for evaluation include A*, Potential Fields (PF), Rapidly-Exploring Random Trees* (RRT*) and variations of the Fast Marching Method (FMM), along with a proposed new method called Updating the Fast Marching Square method (uFMS). The evaluation proposed in this work includes ways to summarize time and safety measures for local path planning techniques. The results in a Lake environment present the advantages and disadvantages of using each technique. The proposed uFMS and A* have been shown to achieve interesting performance in terms of processing time, distance travelled and security levels. Furthermore, the proposed uFMS algorithm is capable of generating smoother routes.

## 1. Introduction

Nowadays numerous applications including data collecting, intelligent transport systems, monitoring of water masses, disaster relief and surveillance, among others, are accomplished by the use of unmanned vehicles, both aerial and/or aquatic vehicles [1]. Unmanned vehicles present important advantages, such as low cost in terms of hardware since they usually have smaller dimensions compared with classical vehicles, and also, they do not need personnel on board since they are self-managed. However, they do require an increase of complexity for the control system. Depending on the environment where they operate in, they can be classified as aerial, underwater, surface or ground vehicles, normally abbreviated as Autonomous Aerial Vehicles (AAV), Autonomous Underwater Vehicle (AUV), Autonomous Surface Vehicle (ASV) and Autonomous Ground Vehicle (AGV) respectively [2]. This work is focused on ASV, which are used in water masses like rivers, lakes and seas for monitoring tasks [3], such as surveillance [4] and bathymetry [5]. When multiple vehicles cooperate each other, they can form swarms, working in a centralized or distributed way to accomplish a target mission efficiently [6]. Furthermore, they can act as a communication repeater or extender in a network of autonomous vehicles [7].

For the development of ASVs, several aspects need to be taken into account; according to [1], the most important features include the control model, the vehicle characteristics and navigation/guidance systems. The latter refers to both localization of the vehicle within the environment and motion planning, which are techniques implemented to plan ahead road maps to accomplish a maneuver or movement goal. While these systems work closely together, methods and techniques are not similar. Although this work focuses on motion planning techniques, it is worth mentioning some localization techniques, which in general are crucial in real-life scenarios, as the ones described in [8], which carefully characterizes the error of the position estimation of an AGV using three different methods, using as ground truth a robotic total station: odometry, extended Kalman filters ([9]) and ultra-wideband localization systems. In respect to motion path planning approaches for ASV, in [10] these techniques have been classified in different levels. At first level, the techniques are divided into global (offline) path planning and local (online) path planning techniques [11]. In global path planning approaches, which are proactive, an initial path is calculated with the available global map information to reduce a target metric like the travelled distance by the vehicle [12]. For instance, calculating the path planning as a sequence of waypoints to visit. On the other hand, the local path planning techniques are reactive approaches, which are used to adapt the initial path calculated by the global planner to unforeseen situations like the appearance of obstacles. Therefore, both techniques can be considered complementary and should be used in the implementation of path planning approaches in real ASV applications.

This work compares several local path planning approaches for monitoring application of the Ypacarai Lake in Paraguay. This lake is known to contain a high concentration of phosphor and nitrogen, in different regions [13]. These nutrients help to the development of cyanobacteria, also known as blue-green algae, which produces toxins that are harmful to humans and animals. The first steps to eradicate this issue consist mainly on monitoring the quality of the water, locating zones or regions containing certain level of parameters that can be considered as dangerous or sufficient enough to produce the algae bloom. These blooms are periodically appearing thorough the years, so constant monitoring is required.

The global path planning algorithm proposed in [12] is a quasi-optimal and efficient way to obtain information about the quality of the water of the whole lake (64 km${}^{2}$). Nevertheless, more information is required to travel through this path, as obstacles, exclusion zones and other aquatic vehicles may appear making the travel not only more difficult but also dangerous. Local path planning provides a way to avoid these situations, accomplishing and modifying the travel that the global path has planned when needed. Since many local path planning algorithms are available, the chosen algorithm must fulfil certain levels of criteria or performance metrics. This work evaluates the most well-known local path planning techniques with the help of a custom made simulator. The evaluation comprises a system that rates the level of movement of a vehicle, evaluating characteristics such as shortest path generation, time consuming rates and safety measures. This proposed evaluation system accomplishes a way to summarize path planning evaluation data in order to help decision-making in motion planning.

This paper continues as follows, Section 2 presents an overview of the local path planning that are studied in this work. Then, Section 3 describes the statement of the problem that includes the path planning problem for the particular case of Ypacarai Lake. Next, in Section 4, a more formal description of the local path planning algorithms is introduced. Section 5 provides the simulation environment and the results of the performance of the local path planning algorithms tested. Finally, Section 6 presents the conclusion and ideas about future work.

## 2. Related Work

Local planners have been studied since the birth of robotics, therefore many researchers kept local planners evolving and also made them more efficient. Nowadays, a large amount of well-studied methods and algorithms exist, including Dijkstra [14], Rapidly Exploring Random Trees (RRT) [15] and Potential Fields (PF) [16].

The goal of this work is to obtain a numerical value that represents the expected performance of each one of the most-used path planning methods and techniques, and in that sense, several path planners are reviewed, studied and implemented in a controlled environment for a surface vehicle. Although there are many reviews on this topic [17,18], the focus in this work is to provide not only a standardized path-planning testing procedure, but also results for an autonomous surface vehicle in a low current speed environment such is the Ypacarai Lake (Paraguay). The most well-known/used algorithms are reviewed, implemented and tested in a controlled environment.

Dijkstra’s algorithm [14] is one of the most used path planning algorithms, it seeks a feasible path starting from an initial position, searching in every direction for the goal position. Using a grid map, the vehicle can implement Dijkstra to find the goal prior to any movement. Then, when the goal is reached by the algorithm, a feasible path is returned and used by the vehicle to get to the goal position. In recent years, practically all works use variations of the Dijkstra’s algorithm [19,20,21], and older ones, like [22], use the algorithms without variations. Dijkstra’s algorithm is described in the literature as a fast, simple path planning method.

The RRT technique is a path planning algorithm, which is based on growing a random tree for searching the goal position. The planner returns an obstacle-free random path. An example can be seen in [23], which uses the RRT algorithm within an ASV to accomplish movements while adopting water collision regulations. This technique is used in fast non-complex environments, where the vehicle must adapt its planned movement, focusing on obtaining paths to the desired goal position and not on the characteristics of the path (i.e., shortest, smoothest, etc.)

PF is a path planner that creates a map that weights each point with a potential value. Generally, it assigns lower values to the positions closer to the goal, so the vehicle should always try to move to the minimum value, eventually reaching the goal position. To plan ahead, gradient descent is the method used to obtain the path. Safe paths can be found as long as local minimum is not present as a obstacle. In [24] they used PF technique to provide local paths to an underwater vehicle, also noting that local minimum represents a problem.

PF offers the possibility of virtual weighting. This can be considered as an advantage as it is supposed that certain zones of the lake will not be as attractive to monitor as others because of the presumed heterogeneous distribution of nutrients, leading to include the study of algorithms that include weighting a map prior to calculating a path.

Fast Marching Method (FMM) [25] is a method that considers both, the map weighting, and goal location by solving the Eikonal Equation of wave propagation. This method extends the functionality of both, the Dijkstra and PF techniques, but is mainly used as offline path planner because of the intense computational power it requires. FMM is used and tested in [26], providing results that accomplish very well the motion planning characteristics for autonomous vehicles. While the mentioned work does not include time calculation in their performance, this information is crucial in local path planning. In that sense, this work evaluates this method and, more importantly, proposes a modification of the Fast Marching Square Method (FMS) to obtain faster results while maintaining other important characteristics of the method by Updating the Fast Marching Square Method (uFMS).

Defined in [27], path planning algorithms can be classified by the necessity of proper environment modelling; the same survey states that, while A* does need an environment modelling, methods like PF and RRT does not need it in order to calculate a path between two points. These methods and their variants are defined in the same work, concluding that path planners with proper environment modelling (i.e., regular grids) are most likely to give optimal results; while the methods without proper modelling are more suitable for real-time requirements.

Other works like [28] emphasize on the problem-space definition and the proven time complexity that each algorithm has, classifying them in related groups (PF, probabilistic, cell decomposition). In the same work, it is recommended that a solution should be chosen if it fits in the characteristics of addressed problem.

This work surveys some of the well-known algorithms in the path-planning literature, detailing the algorithms so they can be implemented in any programming language while also presenting their high level characteristics like complexity and specific safety measures for autonomous surface vehicles. Additionally, this work also takes a step further by simulating and comparing their results in a specific scenario, facilitating the decision making for future researchers in the area.

## 3. Statement of the Problem

### 3.1. The Ypacarai Lake Contamination Problem

**Figure 1:**
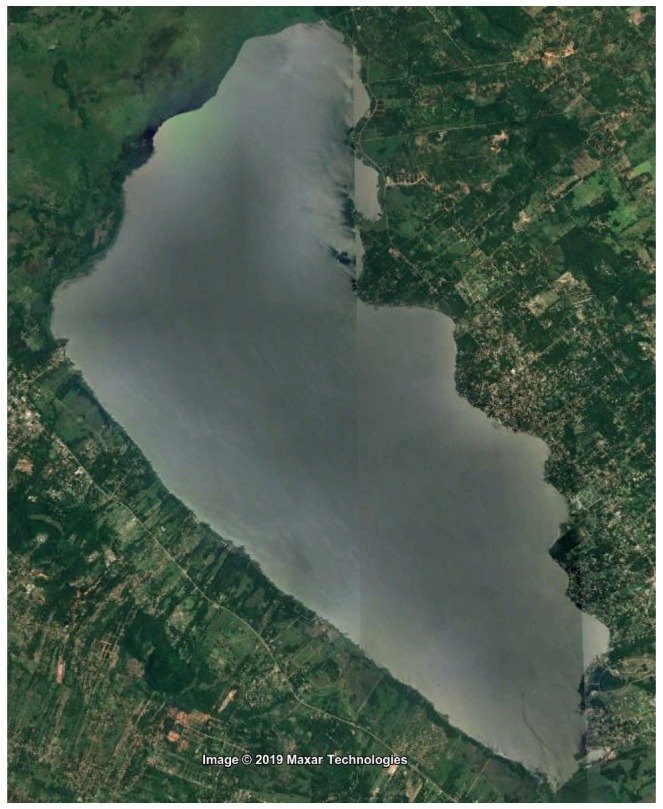
Satellite Image of Ypacarai Lake.

**Figure 2:**
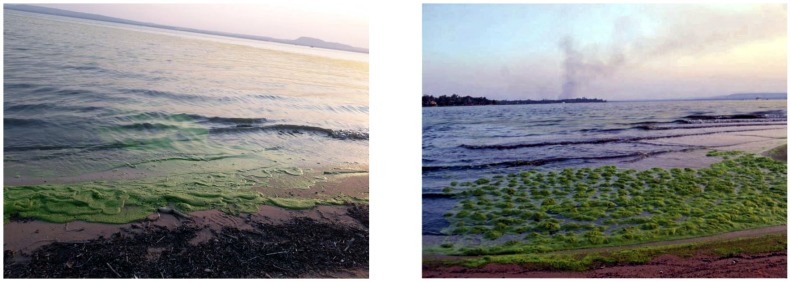
Last appearance of algal bloom at the shore of Ypacarai Lake (February 2019) [30].

The objective of this paper is to perform a study of local path planning techniques and determine the most suitable one for an ASV, particularly a catamaran, oriented for the scenario of Ypacarai lake. Figure 1 shows a satellite image from Ypacarai Lake taken in the end of 2018. It is a shallow lake with an average depth of 1.72 m and a maximum depth of 2.53 m [29]. There is a large amount of population living in the surroundings whose activities have a direct impact on the conditions of the lake. The most notorious evidence of the degradation of the lake is the appearance of algal bloom (Figure 2), normally in the hot season between November and December. Many monitoring campaigns were carried out in the 1980s and more recently in this decade. Samples were taken manually at specific points in the lake and a few fixed monitoring stations were located at the shore. However, having an autonomous vehicle that can find its path through the lake will help in the monitoring tasks of the lake.

The problem that defines local path planning utility for monitoring applications is that, after a global path planning is made, the vehicle should move from one target to another while avoiding any type of obstacles, re-planning safe routes as many times as necessary. Therefore, the local path should be as safe, direct and as fast as possible. Thus, to compare the available methods, these qualitative characteristics should be studied with quantitative measures, whenever possible.

### 3.2. Proposed Global Path Planning

An ideal global path plan for monitoring big bodies of water should contain the least amount of information so that an Autonomous Surface Vehicle (ASV) can be tasked to cover the surface of the body of water, or, at least, a certain amount of surface considered as sufficient. In [12], it is defined and obtained a global path that covers the biggest area possible of the Ypacarai Lake with an ASV, using Genetic Algorithm (GA). For this purpose, 60 waypoints or intermediate goal nodes are defined, that the ASV should visit at least once (similar to the Travelling Salesman Problem (TSP). Then, it obtains via a GA, a visiting order so that the ASV covers the biggest possible surface of the lake in one run. Furthermore in [31] they proposed the use of Eulerian Circuits instead of Hamiltonian Circuits in TSP for solving the global path planning problem. The difference of this latter approach with the TSP is that the ASV can visit a waypoint more than once time. The resulting 60 waypoints list and the visiting order can be fed to a local planner, the path planned, obtaining a result ready to be executed.

Though an ASV could travel directly between waypoints, some form of obstacle avoidance and/or path modifications that considers vehicle and environment constraints should be planned ahead, to have an estimated result prior of executing the movement. Furthermore in large maps, it is impossible to predict dynamic obstacle presence and pinpoint the exact location of them beforehand. Therefore, local path planning is the tool used to obtain this result.

### 3.3. Local Path Planning

As defined in [32], Local Path Planning is a planning method that will be responsive to obstacles and changes of the environment, whether it is dynamic or unknown. In addition, local path planners are known to calculate short paths, usually in the vehicle line of sight and in real time, updating the direction of the vehicle heading if necessary. The essence of the local path can be applied to a more broad group of path planners that track the changes of the scenario, and ask for an updated planned path whenever the current one is no longer feasible. Additionally, this method helps to obtain more information about the environment by storing the changes that the vehicle senses. This work studies some of the standard local path planning algorithms and compare them in order to confidently select a proper path planning technique to be used as local path planner in an ASV that studies the water quality of the Ypacarai Lake.

## 4. Local Path Planning Algorithms

This section presents a summary of the main local path planning techniques found in the literature. This summary includes an overview of each method together with a pseudo-code and a block diagram describing its operation.

### 4.1. Pure Pursuit

Although basic *Pure Pursuit (PP)* is not a local path planning algorithm *per se* because it only tries to reach a certain point without having a reactive behavior, it is used as starting baseline model for comparison purposes. In fact PP is a tracking algorithm that calculates the curvature of the movement of the vehicle from its current position to some goal point [33]. The algorithm finds some ahead point that it is located along the path. However, in our case, no obstacle avoidance is made. Therefore, the ASV moves directly towards each beacon or waypoint. Equation (1) shows how the velocity *v* is calculated having a *k* gain, a target or goal, and a current position pose of the ASV. Using this equation, Algorithm 1 is implemented to have the ASV driving from each beacon (waypoint) to another.

(1) $$v=k\frac{target-pose}{\mathrm{norm}(target-pose)}$$

**Algorithm 1:** Pure Pursuit

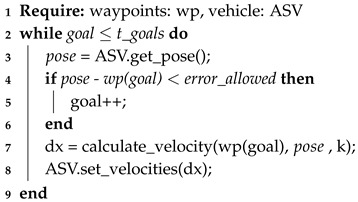



### 4.2. A* Planning Algorithm

A* is a local path planning algorithm that adds a heuristic function to the well known Dijkstra’s algorithm. In the classical Dijkstra’s algorithm, it searches a route between two positions by examining in each iteration the neighbors of a parent node, i.e., all nodes connected to the parent node or position, and considering each neighbor as a future parent node. This process is repeated until the goal node is found; then, a sub-function is called to find a road map with the information of the neighborhoods found. In contrast, A* algorithm adds a priority queue, the Frontier List, which prioritizes the examination of new nodes closer to the goal and/or with a lower cost. In this algorithm, the cost is defined by the vehicle and/or environment restrictions and the desirable movement. Therefore, higher costs can be assigned to nodes next to obstacles or some types of movements can be forbidden (i.e., crossing diagonally if there are obstacles in between). These costs are summed up and then assigned as the priority of each new node. Thus, lower costs nodes have higher priority in the queue and have higher chance to be the next studied or observed node. As a result, the computational total time is reduced drastically, while maintaining the convergence and security level that the Dijkstra algorithm offers. The complexity is known to be O(N) with an optimal heuristic approach.

**Figure 3:**
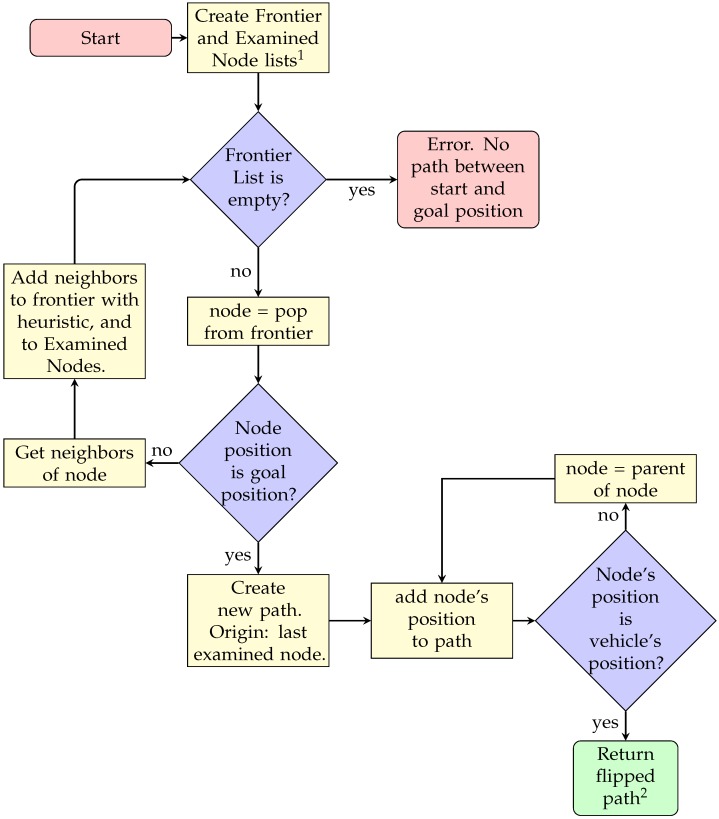
A* sequence diagram. 1: Both lists include the vehicle’s initial position as node. 2: The path should go from end to start.

As observed in Figure 3 the algorithm follows a three-decision block sequence. The first two are used during the creation of the map, or node graph. During these steps, the Frontier List acts as a queue, where a node is popped and examined, and added to the Examined Node List. All of the non-examined neighbors of this node are added to the Frontier List, for future examinations. Then, the last decision block is found during the creation of the path, using the Examined Node List. A pseudo-code is presented (Algorithm 2), where a priority queue of the next possible nodes to be examined is added to finally arrive and examine the goal node. A path is then generated by calculating the lower cost nodes that connect the goal and start nodes.
**Algorithm 2:** A* planning algorithm
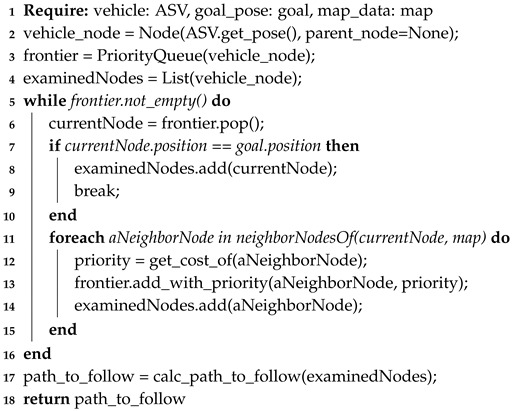


By modifying the Pure Pursuit runtime algorithm (Algorithm 3), the A* is implemented by adding this planning every time the ASV reaches a goal. As observed in Algorithm 3, no major changes were made, but A* is used for path planning. The algorithm is executed as follows. First, the goals are the waypoints obtained from the global path planning. Between these waypoints, the p_planner (line 4 in Algorithm 3) is executed (line 12 in Algorithm 3), which in this case is the A* algorithm. The output of this algorithm are intermediate points that are called sub_goals. The Pure Pursuit now tracks the direction according to these sub_goals (line 16 in Algorithm 3) instead of the goals. This procedure is repeated until visiting all the waypoints.
**Algorithm 3:** Runtime Algorithm, including path planning
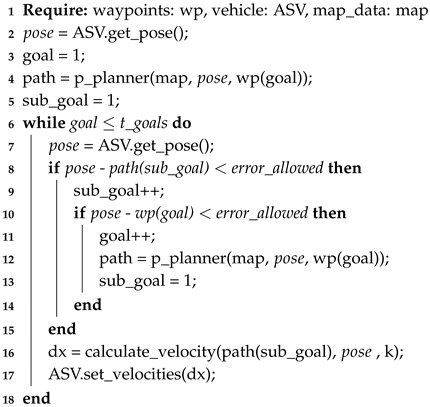


Algorithm 3 will be used as the base structure to build the following local path planning techniques, only modifying the part corresponding to the p_planner.

### 4.3. Potential Fields Algorithm

Potential Fields (PF) path planners are widely used in robotics. The basic idea of PF consists of defining a potential value to every point on the region by generating two types of fields, a repulsive field and an attractive field. PF path planning is inspired by nature, more specifically, the attractive and repulsive fields generated by electromagnetic fields. In [16] two main functions are defined, Equations (2) and (3). Then both $U_{atr}$ and $U_{rep}$ should be added to find a potential value for each point x,y within the region.

(2) $$U_{atr}=\frac{1}{2}\times k_{a}\times pdist\left(pose,goal\right)$$

(3) $$U_{rep}=\frac{k_{r}}{pdist(pose,obst_{i})}$$

**Figure 4:**
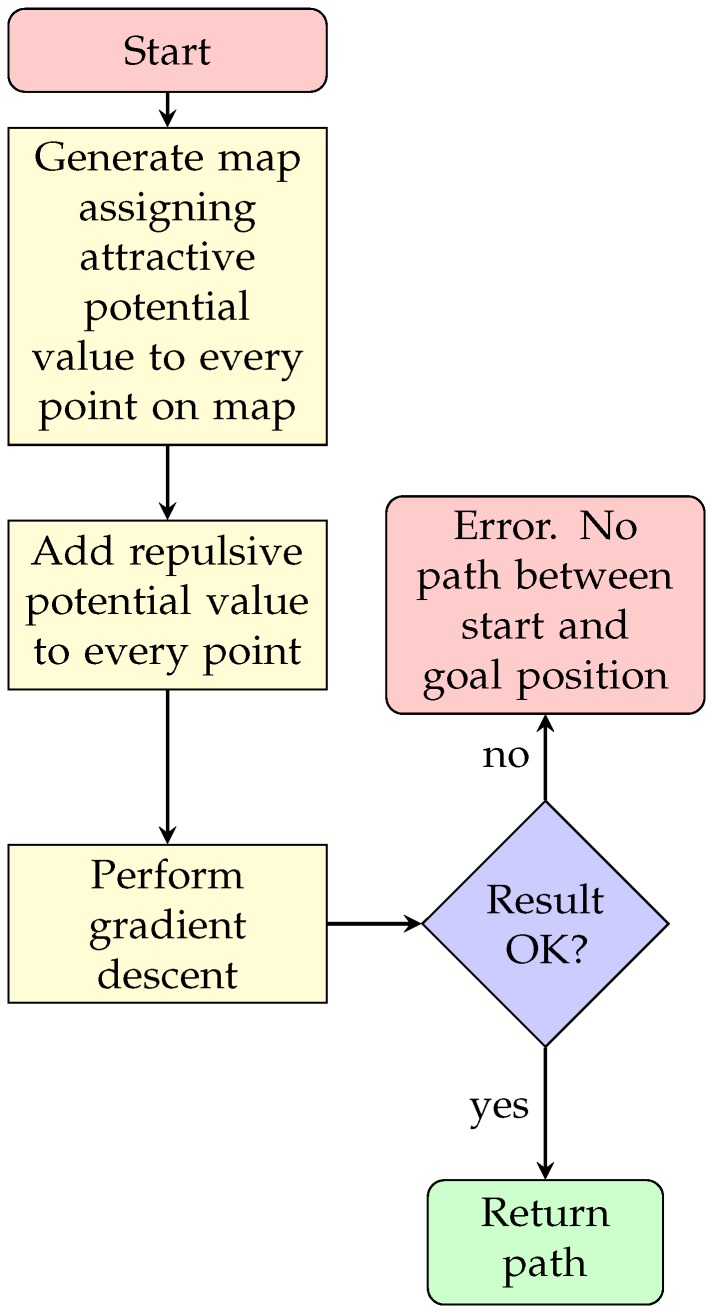
Potential Fields sequence diagram.

**Figure 5:**
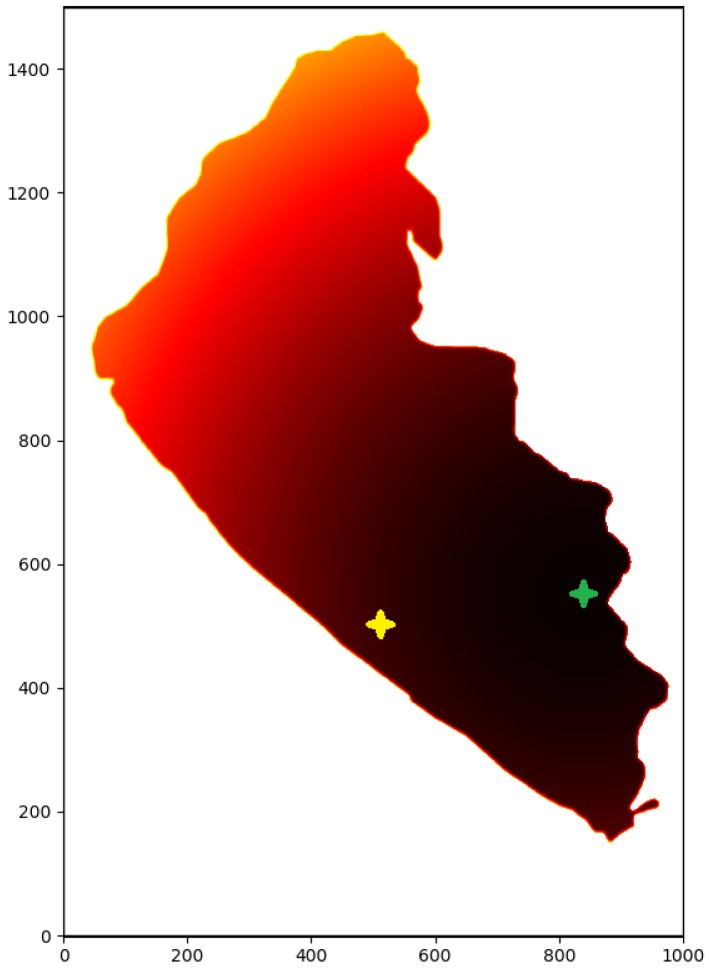
Generated potential map for a goal.

In these equations, $k_{a}$ and $k_{r}$ are attractive and repulsive constants, and pdist measures the Euclidean distance between the position being examined (pose) with the goal and the obstacle *i* ($obst_{i}$) respectively. A procedural way for obtaining a path, can be via calculating the gradient descent of the map. The general sequence diagram for PF path planning can be observed in Figure 4. A result of calculating a potential map can be observed in Figure 5, where the goal was to assign small potential value to the regions around the goal position (green star), and high potential values to regions where the vehicle should not travel. The white color corresponds to prohibited zones or positions, like terrain, or maybe even obstacles. Additionally, the yellow star corresponds to the starting point, which serves no purpose while calculating the potential map.

Once a potential map is generated, gradient descent can be performed from start to goal position in order to generate a path and, if feasible, the path is given to the ASV. When the ASV reaches a target, the next map is generated and used by the ASV. The complexity of this approach O(Nlog(N)) According to [28], and since it involves the map generation and its calculation via gradient descent.

### 4.4. Rapid-Exploring Random Trees* Algorithm

One of the most popular sampling-based planners is the Rapid-Exploring Random Tree (RRT) [15]. The RRT algorithm grows a tree with nodes and branches connected to each other. These nodes are created randomly and then added to the tree until a random node connects to the goal target. In order to improve the results or creation of random nodes, RRT* [34] has been proposed as a modified version that creates nodes within the neighborhood of a target pose, before adapting this node to the tree. Because of the direct dependency of distance, the proven complexity is the same of A*, O(N).

**Figure 6:**
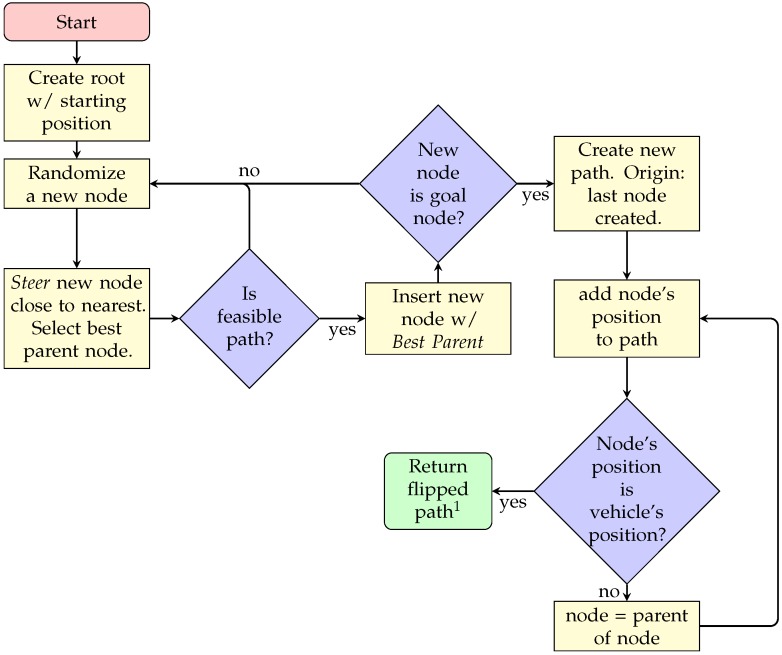
RRT* sequence diagram. 1: The path should go from end to start.

A sequence diagram for this method is presented in Figure 6, which is fairly more complex than the previous path planners presented. This is because the method is broken into two differentiated algorithms, excluding the main algorithm, each of them accessed depending of the decision made by the main algorithm.

The main algorithm of RRT* can be found in Algorithm 4, then sub functions for steering and choosing a parent in Algorithm 5 (line 7 in Algorithm 4) and Algorithm 6 (line 10 in Algorithm 4), respectively. In the main algorithm, a tree τ is initialized, containing as main branch or root, the starting point as a node. Then the procedure iterates by sampling or creating a new node within the region $R^{n}$. After randomizing a sample node *P* (line 5 in Algorithm 4), the algorithm finds the nearest node from tree τ (line 6 in Algorithm 4) and steers the sampled node to the nearest node, if needed (line 7 in Algorithm 4). This steering is done in Algorithm 5, where the distance between $p_{rand}$ and $p_{nearest}$ is checked to be within certain maxStepDist distance (line 3 in Algorithm 5). If not, a new point $p_{steer}$ is generated by defining its direction to be the same as the vector difference between $p_{nearest}$ and $p_{rand}$, and the magnitude equal to maxStepDist; this $p_{steer}$ is assigned as the new $p_{rand}$. Back in the main algorithm, it is checked if there is a feasible route between $p_{nearest}$ and $p_{rand}$, after that. The function chooseParent (line 10 in Algorithm 4) obtains the parent with the lower cost and, as [34] suggests, the new node should be connected to the best parent using the Algorithm 6, which calculates the lower cost of connection to a parent and returns this parent. The same work defines the cost of a new node as the distance between the new point and a node of the tree. Finally, the new node is added to the tree and connected to its best parent together with the calculated cost. Afterwards, the procedure repeats until a random node is located within the position of the goal or desired position. Then, the path should travel from the last created node to the starting node. In order to improve results, the randomized new node could be randomly sampled to be the goal position. RRT is one of many path planners that returns an “any angle path”, meaning that the vehicle is able and instructed to travel in any direction, unlike the previous planners that may travel only in several discrete directions.
**Algorithm 4:** Main algorithm for RRT*
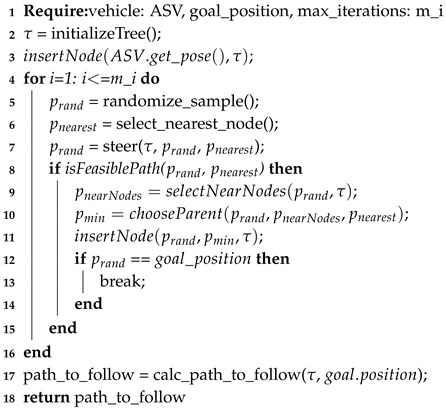


**Algorithm 5:** Rapidly-Exploring Random Trees (RRT) steering function.

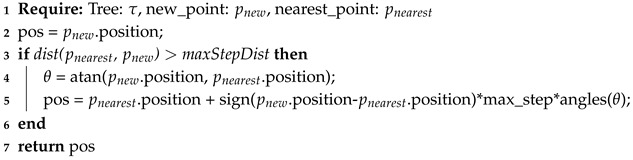



**Algorithm 6:** Obtaining the best parent node for a new node

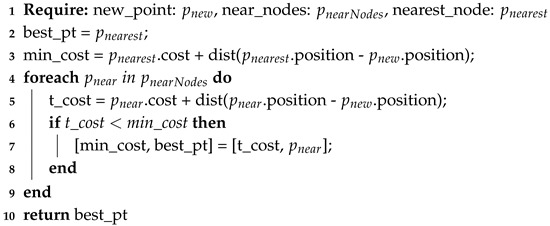



### 4.5. Fast Marching Method and Variations

#### 4.5.1. Fast Marching Method and Fast Marching Square Method

The Fast Marching Method [25] is an offline path planning algorithm, which solves efficiently the Eikonal Equation (Equation (4)), computing the propagating fronts of a wave.

(4) |∇T(p)|V(p)=1

where T(p) represents the arrival time to point *p*, V(p) is the propagating velocity at point *p*, and ∇ the gradient. The FMM algorithm proposes to discretize Equation (4) to calculate T(p)∀p(x,y) by using a map *M*, which contains all points $p\in R^{n}$, and defining Δx and Δy as minimun possible movements.

The last description can be used to define von Neumann’s neighbors of a point *p* as (x+Δx,y), (x−Δx,y), (x,y+Δy) and (x,y−Δy). Finally, according to [26], the value of T(p) can be found using the following equations:

(5) $$T_{1}=min\left(T_{\left(x+\Delta x,y\right)},T_{\left(x-\Delta x,y\right)}\right)$$

(6) $$T_{2}=min\left(T_{\left(x,y+\Delta y\right)},T_{\left(x,y-\Delta y\right)}\right)$$

(7) $${\left(\frac{T_{\left(x,y\right)}-T_{1}}{\Delta x}\right)}^{2}+{\left(\frac{T_{\left(x,y\right)}-T_{2}}{\Delta y}\right)}^{2}=\frac{1}{{\left(V_{\left(x,y\right)}\right)}^{2}}$$

The solution of these equations is given by:

(8) $$T_{\left(x,y\right)}=\left\{\begin{array}{cc} T_{1}+\frac{1}{V_{\left(x,y\right)}}, & \mathrm{if}\;T_{2}\geq T\geq T_{1}.\\ T_{2}+\frac{1}{V_{\left(x,y\right)}}, & \mathrm{if}\;T_{1}\geq T\geq T_{2}.\\ Solution\;of\;Equation\;(7), & \mathrm{if}\;T>max(T_{1},T_{2}). \end{array}\right.$$

This equation is known as the Cost Update function, and will be the function that calculates the time of arrival or cost update for every point in the region. In the FMM algorithm, the velocity matrix $V_{\left(x,y\right)}$ takes binary values (0 or 1), where its dimensions correspond to the map *M* dimensions. The Δ step is defined to be the same in both, *x* and *y* directions, and equal to the unitary value to facilitate the solving of the Equations (5)–(8). In order to determine the correct values in an iterative way, the order of obtaining these values must coincide with the propagation of this hyper surface. Therefore, the values must be calculated from a starting point outward. Thus, it can be said that the resolution of the FMM is similar to the resolution of the Dijkstra but in a more continuous way.

**Figure 7:**
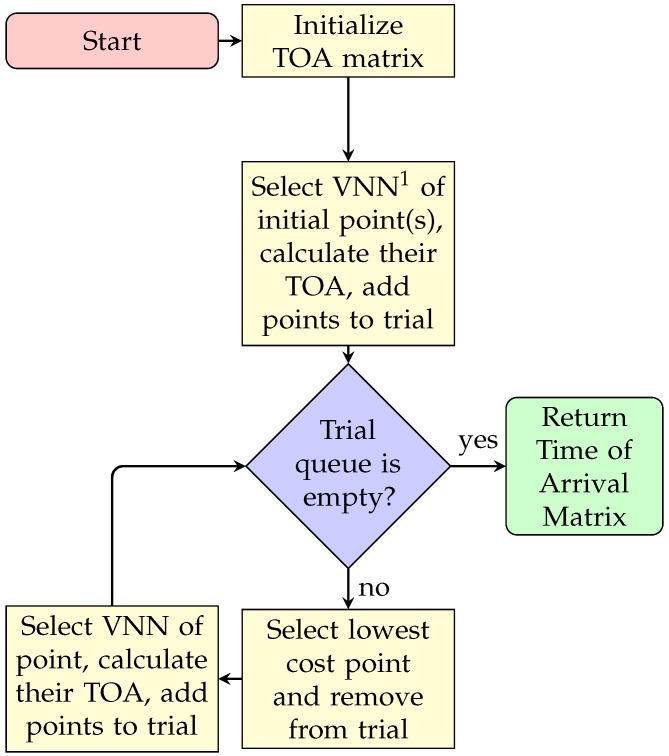
FMM: Procedure to find Time of Arrival (TOA) matrix. 1: Von Neumann’s Neighbors.

Figure 7 shows the sequence diagram to obtain the Time Of Arrival (TOA) matrix. This algorithm requires an initial point (or points) and a velocity map, and returns the TOA matrix, which can be considered as the final matrix to calculate the route.

This approach is presented in Algorithm 7, taking into account that the time of arrival $T_{p_{0}}$ to the starting point is always zero (line 3 in Algorithm 7). Then between lines 5–9 a trial queue is built with the VNN of the starting point p_start, and from there all the arrival times of the remaining points of the map are calculated with the cost_update() from Equation (8) (line 19 of Algorithm 7. The algorithm also checks if there are undefined points with cost→∞ (line 13 in Algorithm 7).
**Algorithm 7:** Solving Fast Marching Method (FMM) Time of Arrival matrix.
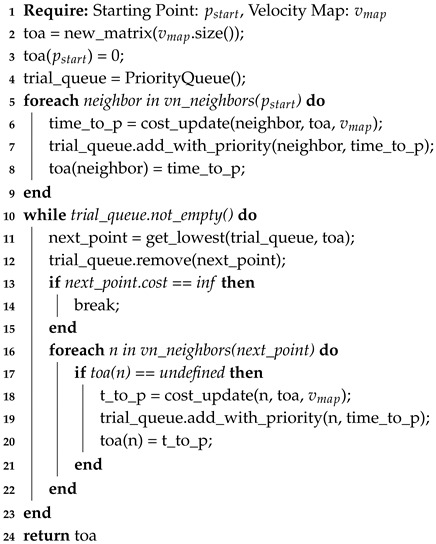


**Figure 8:**
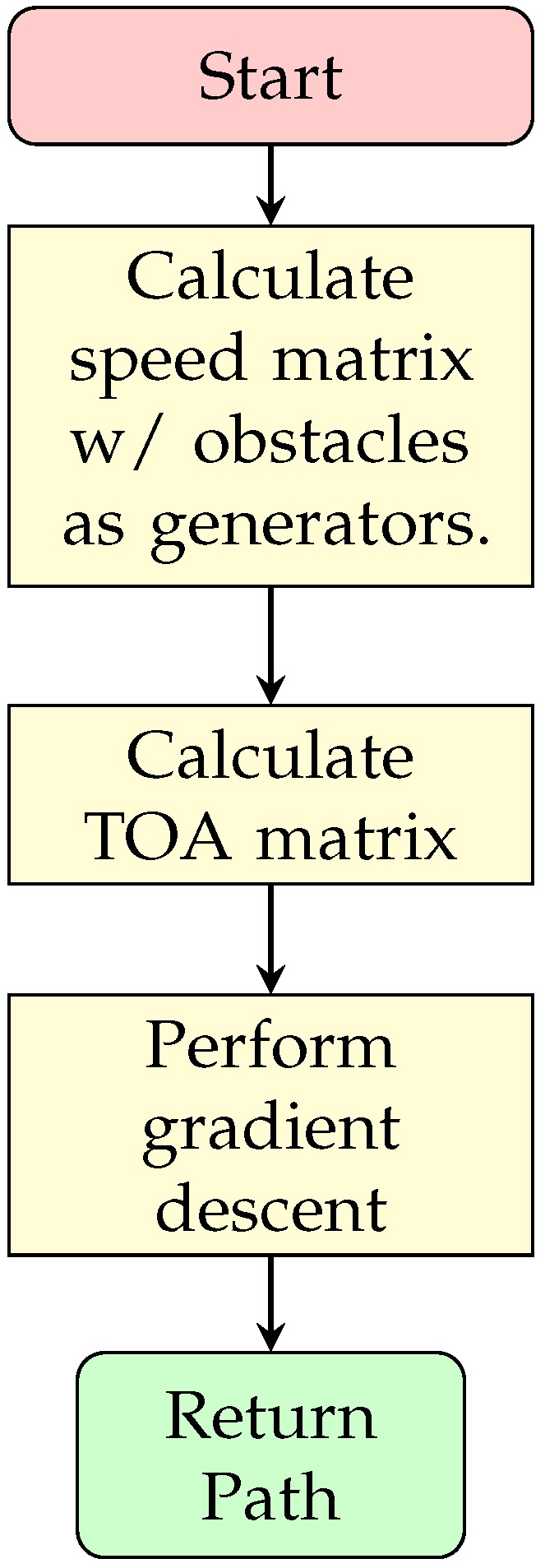
FMS sequence diagram.

Fast Marching Square Method (FMS) is a variation of the same method that performs the FMM twice. The procedure can be observed in Figure 8. The Algorithm 8, extracted from [26], shows that the same method is used twice, the first time it uses the Cost Function to calculate the velocity map (using Equation (8) with the TOA matrix as result, line 2 in Algorithm 8); setting any present obstacle as generators or initial points. So Algorithm 7 must be prepared to receive as initial state more than one point. Then, from those points the velocity map is generated (line 3 in Algorithm 8). With the calculated velocity map, the algorithm proceeds the same as the usual FMM, assuring a modified velocity at all positions that are in the presence of obstacles (line 4 in Algorithm 8). Most likely the map will be different from the binary map used in the previous method.
**Algorithm 8:** Fast Marching Square Method.1 **Require:** Initial Map: *M*, Starting Point: $p_{start}$, Goal Point: $p_{goal}$ 2 V = calculate_speed_matrix(*M*); 3 OB = perform_fmm($p_{obstacle_{i}}$, V); 4 TOA = perform_fmm($p_{start}$, OB); 5 path = gradientDescent (TOA, $p_{start}$, $p_{goal}$); 6 **Return:** path;

There is a different time complexity for these methods because of map calculations; while FMM requires a single map calculation, FMS requires two, so the complexity of these methods are O(Nlog(N)) and $O(N^{2}\log\left(N\right))$ respectively.

#### 4.5.2. Proposed uFMS

Updated Fast Marching Square Method (uFMS) is proposed in this work to improve the efficiency and effectiveness of the FMS by using local methods of updating the generated maps. As the original method, it is divided into two fundamental parts but it is focused on drastically decreasing the total time to generate a route by updating only zones or regions that present new information.

The main problem to mitigate in FMM or FMS is that the entire TOA matrix should be calculated even if the difference between some previous map and a new one is small (new obstacles, new free or occupied zones). To solve this problem, the uFMS algorithm saves the map previously generated by the FMS method, and updates both matrices (velocity and TOA), exclusively in a defined region that contains the modification of the map.

**Figure 9:**
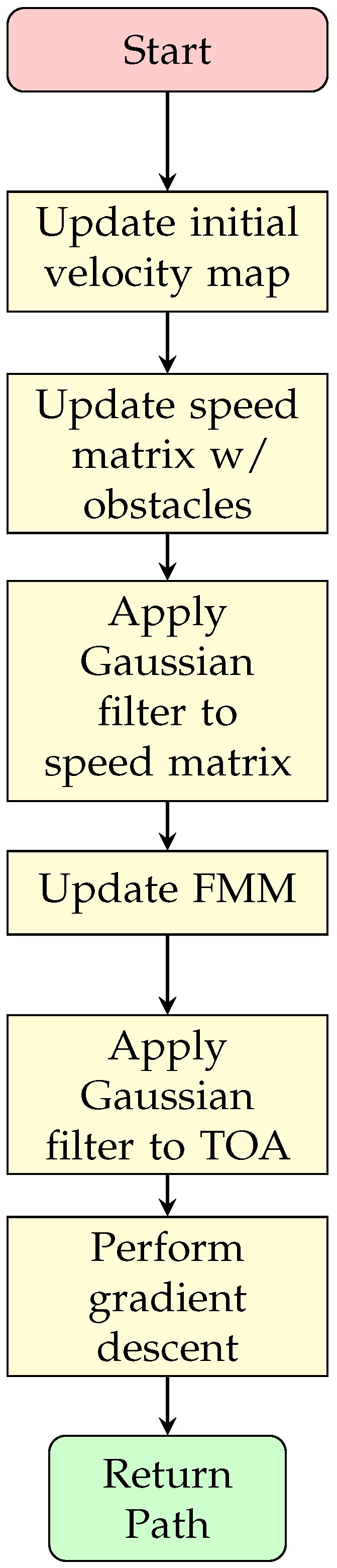
uFMS sequence diagram.

As a first instance, the region to be modified is defined by a position to update $p_{new}$ and an update radius ρ. For this, two vectors containing the horizontal and vertical distances $d_{x}$ and $d_{y}$ from $p_{new}$ to all points *p* of *m* are generated. Then, a radial mask is generated with Equation (9).

(9) $$mask=\left\{\begin{array}{cc} 1, & \forall \;d_{x}^{2}+d_{y}^{2}\leq \rho^{2}.\\ 0, & \mathrm{else}. \end{array}\right.$$

which provides the values that are at a distance less than or equal to a defined radius, forming a radial mask. This mask is used as base to determine which values should be recalculated in the TOA and velocity matrices, redefining these values as undefined. The sequence diagram can be observed in Figure 9. The Algorithm 9 also shows the procedure. It is worth noting the similarity with the FMS (Algorithm 8), but the requirements change as the information of a previous map is necessary. The alteration point and a radius are also added and considered for solving the algorithm. Moreover, instead of the usual calling of update_fmm() function, an update_fmm() function (found at lines 3 and 5) is used, which adds the functionality of modifying strictly the previous map through the use of the Equation (9). It is also important to note that a function is added to apply a Gaussian filter (lines 4 and 6 of Algorithm 9), to smooth the result, which is necessary to improve the costs calculated for arrival times and the response of the descending gradient. The update_fmm() function is presented in Algorithm 10, which presents some slight crucial modifications.
**Algorithm 9:** Updating Fast Marching Square Method.1 **Require:** Starting Point: $p_{start}$, Goal Point: $p_{goal}$, Information from last map: (TOA, OB, V), Modification Point: $p_{new}$, Modification Radius: ρ 2 update_velocity_map(V, $p_{new}$); 3 OB = update_fmm(OB, V, $p_{new}$, ρ); 4 OB = apply_gaussian_filter(OB); 5 TOA = update_fmm(TOA, OB, $p_{new}$, ρ, $p_{start}$); 6 TOA = apply_gaussian_filter(TOA); 7 **return** path, TOA, OB, V

**Figure 10:**
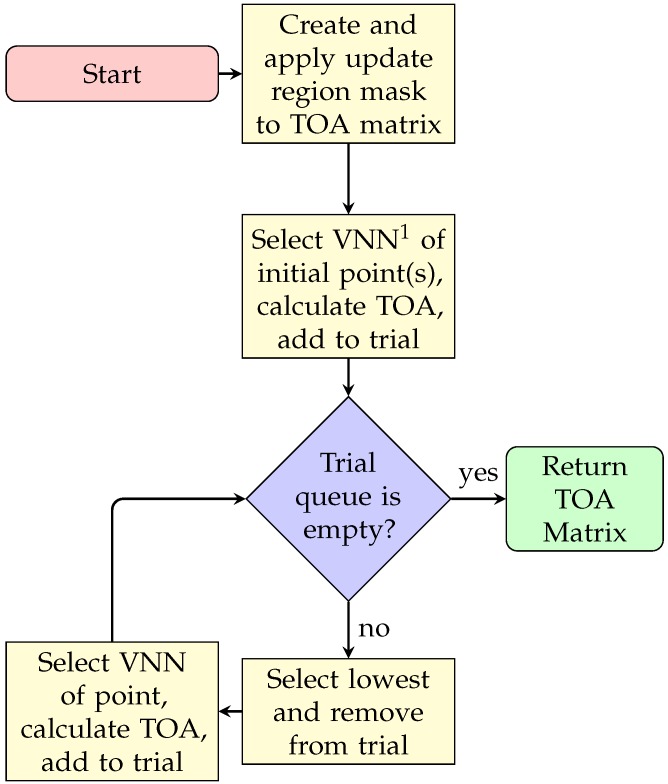
uFMS: Procedure to update TOA matrix.

In this algorithm, lines 3–9 impose the restriction that the map must obtain the new values only within the desired zone. The rest of the algorithm is equal to Algorithm 7. All these changes can be observed in the sequence diagram in Figure 10. Since FMS and uFMS in static conditions will provide the same results, the result of running this algorithm will produce the same output.
**Algorithm 10:** Updating procedure in Updating Fast Marching Square Method (uFMS).
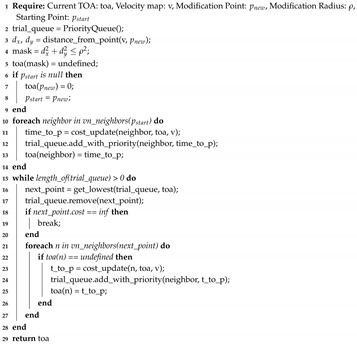


## 5. Evaluation of Local Path Planning Algorithms

Validation of motion planning techniques are a main concern nowadays, because of the existence of a considerable amount of path planning algorithms in the state-of-the-art. In that sense, this works presents a formal generalized way to obtain the performance value of some path planning techniques. This is achieved by defining the vehicle, the environment in which the vehicle will move and a measuring system.

The first approach on evaluating robotics procedures is through simulation. Although it does not represent the real world, it provides a safer, cheaper and controllable way of testing data. For this reason, in this work, the algorithms are tested and measured using a simulation environment, based on the Robotarium Framework [35]. The Robotarium Framework provides a 2-D environment and simulated 2WD-vehicles; both of these characteristics were ported to the developed simulator in our research laboratory. In addition to the characteristics previously mentioned, control systems and measuring systems were added to obtain a proper testing environment. The simulation environment is defined in Section 5.1, while the simulation procedures and the simulation results are defined in Section 5.2 and Section 5.3, respectively.

### 5.1. Simulation Environment

All path planning algorithms require data of the world space in which the vehicle moves. In this work, this data is comprised in a *m* by *n* matrix, simulating a 2-D environment (Euclidean plane), where the vehicle is always located within a point $p\in R^{2}$. Furthermore, the most common method of Cell Decomposition is used as all $p_{i,j}$ elements of the world space representation matrix are regular in size, shape and arrange. This decision facilitates the use of any image since a y,x pixel of the image will correspond directly to a $p_{i,j}$ element of the matrix.

Particularly, an image of the Ypacarai Lake in black and white was obtained using an online tool [36], resulting in a reliable representation, where a black pixel corresponds to land and a white pixel to water. This is generalized where white pixels represents free from collision or reachable positions and black represents obstacles, unreachable zones, prohibited areas, etc. This image has a dimension of 1000 by 1500 pixels, where the distance between the center of adjacent pixels measures roughly 10.33 m. This distance is a very important value because it defines the resolution of the framework, which inherently affects the level of adaptability of the studied methods. This statement will be discussed in the following paragraphs, as it also depends on the kinematics of the vehicle.

It is worth noticing that the vehicle could be located anywhere in the map within a pixel *p*. This is because the vehicle is represented as a point in this context, and due to the fact that the real ASVs to be tested have small dimensions (2–4 m) compared to the pixel size. In the simulation environment, this vehicle is capable of moving according to a set of constraints and configurations that properly models a real ASV. After a path is planned, the ASV obtains the direction to travel to a specific goal position. This direction consists on a 2-dimensional vector that contains the information of the desired velocity $\vec{V}$.

As all real systems cannot accomplish to reach desired values perfectly, the simulated vehicle takes this into account by assigning a function that maps desired velocities to modelled “real” values. The simulated vehicle corresponds to a catamaran, a double-hull boat with separated propulsion systems, one on each hull. This configuration allows the vehicle to rotate on its center, which corresponds to unicycle dynamics. A proper model definition is available in [37]. The unicycle model uses the information of the vehicle desired velocity as well as its orientation (rotation angle with respect to a horizontal line, θ), obtaining a linear velocity *v* and an angular velocity ω calculated with the set of equations Equations (10)–(12) where $k_{v}$ and $k_{\omega }$ correspond to velocity limits.

(10) A=(cosθ,sinθ)

(11) $$v=k_{v}\left(A_{1}{\vec{V}}_{x}+A_{2}{\vec{V}}_{y}\right)$$

(12) $$\omega =k_{\omega }\frac{\arctan(-A_{2}{\vec{V}}_{x}+A_{1}{\vec{V}}_{y},v)}{\frac{\pi }{2}}$$

This v,ω velocity pair is suitable to approximate the movement of an ASV, and it should be fed to a movement simulator environment, that updates the position of the vehicle after a certain Δt time-step. In this particular setup the time-step is 1/12,551 s, and the linear velocity equals to 0.033 pixels/time-step. These are the default values found in the Robotarium framework.

A fully-functional simulator has been developed in Python which also provides visualization and some defined measuring performance parameters [38]. The simulator also updates the state or position of the simulated ASV according to the angular and linear velocities updated in each step, and to kinematics of the vehicle. Furthermore, the simulator comprises a kinematics system to update the position of the vehicle, a localization system, including a map and a simulated LIDAR system, a visualization system, to produce visual output, and an auxiliary evaluation system, for evaluation purposes. The kinematics system includes simple functions to calculate the position of the simulated vehicle, taking into account a model of surface vehicles with their respective maximum velocities, turns and constrains. On every step, this system calculates and updates the position of the vehicle accordingly. The simulator map requires an image file, preferably monochromatic, in which the free zones and obstacles zones are clearly differentiated, (i.e., black and white tiles) and information about which tiles are obstacles. The simulator stops if the vehicle is not located in a free zone.

### 5.2. Simulation Procedures

As this work pursues the selection of an appropriate Local Path Planning technique for monitoring application of the Ypacarai Lake, a simple model of the lake was obtained and fed as an image into the simulator, with shores as obstacle zones, and water as free zones. The mission of the simulated ASV was to travel from one waypoint to another using the 60 waypoint system as in [12], rearranged to fit the model of the lake. These beacons were placed near the shore, inside the lake, considering the transmission range of wireless RF technology modules (e.g., Xbee [39]), using the procedure shown in [38], so that distances between adjacent beacons were equal or lesser than the maximum range (1000 m). The purpose was that, in a real application, these waypoints formed a multi-hop network so the ASV could transmit the sensed data to a base station. These waypoints were used as goal positions and the mission was considered as complete whenever the vehicle visited all 60 waypoints at least once.

After this initialization, the main Algorithm 3 ran and the position of the ASV was updated right after the ASV.set_velocities(dx) function (line 17), so the simulator continuously checked whether the ASV reached the current goal, and responded accordingly.

In order to compare methods, each of the path planners (A*, PF, RRT*, FMS and uFMS) were called in lines 4 and 12 of Algorithm 3 for 10 different possible global path solutions. For the sake of comparison, several performance parameters were measured, such as the time of path planning, the total simulation time, the difference in the distance with the ideal path, and the security levels. Then, each of these values were normalized and equally weighted in order to provide a grade in a scale from 0 to 100, being the best technique the one with the greatest number.

The measured parameters by the evaluation system included the following:

1. Time of Path Planning: measured whenever the vehicle started to plan a new path until the calculation was done and a valid path was obtained. This measure shows the path planner time complexity. Ideally, path planners used a little amount of time to calculate a path, and as Pure Pursuit algorithm did not compute any path planning, it can be used as standard or ideal time (0 s). As the distance varied between waypoints, the average values were calculated.
  Equation (13) was used to obtain a normalized and weighted value. In this equation, $t_{pp}$ refers to the mean time of path planning, *w* to a weighting constant and $t_{ppmax}$ the maximum allowed value of mean time of path planning. It is important to emphasize that the weighting constant was equal to 25 as there were four evaluation parameters, giving a maximum total of 100 when added. Additionally, the maximum allowed time was defined to keep the values in the same order of magnitude.
  (13) $$t_{wpp}=\frac{w}{t_{ppmax}}\times \left(t_{ppmax}-t_{pp}\right)$$
2. Total Simulation Time: As every path planner generally generated a different path, the total simulation time $t_{st}$, including the first criterion and also the time it took to travel through the planned path, should also be considered, since the planner could return an obstacle-free path that took longer time which is not optimal. The function for obtaining this value (Equation (14)) slightly varies from the previous one because there is a minimum total simulation time given by the solution of the Pure Pursuit algorithm, which is different from zero. The maximum value is also different as it represents a random maximum simulation time to accomplish the objective.
  (14) $$t_{wst}=\frac{w}{t_{stmax}-t_{stmin}}\left(t_{stmax}-t_{st}\right)$$
3. Difference in Distance Driven: Because of specifics of the work in [12], the real traveled path should be as equal as possible as the path calculated by the GA global path planner, in which direct routes from waypoint to waypoint were assigned. Environment modeling does contribute on generating different routes than those of a straight line, but path planners generally do not provide this straight, direct routes between two points because of procedures. Measuring the difference of the distance between two points $dist_{i}$, and the real distance traveled between the same points $dist_{{travel}_{i}}$ is important because it provides a way to measure the change of global path planning versus the executed path.
  A way to obtain a weighted value of this measure is calculating first the average value between the *n* extra traveled distance (difference between traveled distance and calculated distance for every route *i* in *n*) and calculated distance between points for every path, Equation (15). Afterwards, the weighted can be calculated with Equation (16), with a maximum $ddif_{wmax}$ allowed.
  (15) $$m\_dif=\frac{\sum_{i=1}^{n}\frac{dist_{{travel}_{i}}-dist_{i}}{dist_{i}}}{n}$$
  (16) $$ddif_{w}=\frac{w}{ddif_{wmax}}\times \left(ddif_{wmax}-m\_dif\right)$$
4. Security Level: As the path planning algorithms generally do not take into account the dynamics of the vehicle, this criterion rated the level of security, or how secure it is to execute the planned path. The generated paths were rated observing generated path and assessing its quality through a weighted value according to the items below:
  - Level 5: The path contains no drastic turns or maneuvers that an ASV cannot handle, the path avoids obstacles easily and prevents the ASV from collision.
  - Level 4: The path generally contains maneuvers that an ASV can handle and execute, all obstacles are avoided but the ASV can travel very close to them.
  - Level 3: The path contains drastic turns and maneuvers that an ASV cannot handle easily, obstacles are avoided but the ASV can travel very close to them.
  - Level 2: The path contains turns and maneuvers that are very hard to be executed on an ASV, obstacles whatsoever are avoided.
  - Level 1: The path contains maneuvers that are not possible on an ASV, crosses an obstacle or does not take into account any obstacle present.

The security level is the only criteria adopted that is not generalized to any kind of vehicle, because it heavily depends on the vehicle dynamics. The security level should be previously defined for every type of vehicle, taking into account the safeness need and the vehicle dynamics.

With the procedures and criteria properly defined, the simulations were executed. The simulator was written and run in Python 3.6.5, in a Windows 10 x64 OS, and in a machine with the following specifications: CPU Intel Core i7-7700HQ @ 2.8 GHz and 8 Gb RAM memory. The simulator as well as the path planners and examples codes can be found in [40].

All path planners used the same set of waypoints in each simulation, and visited each of them in the same order. The goal of the ASV was to visit each waypoint once, and when one was visited, a path to the next waypoint was calculated and the calculation time measured. In every iteration, the distance traveled in a delta time step was stored and added to the total. Table 1 and Table 2 show the parameters used in the simulation environment and the configurations parameters used to calculate the performance of the path planners according to the aforementioned performance metrics.

### 5.3. Simulation Results

Before any specific discussion on the proposed methods, the discussion focuses on the parameters for both Table 1 and Table 2. A total of 60 simulations were run and all of them achieved the goal of visiting the waypoints in the established order. As the error allowed (recall Algorithm 3) was the same for all methods (error_allowed=0.01pix), and the same kinematics model and parameters were used, the comparison between algorithms is impartially devoted to the qualitative-quantitative characteristics of each method.

**Figure 11:**
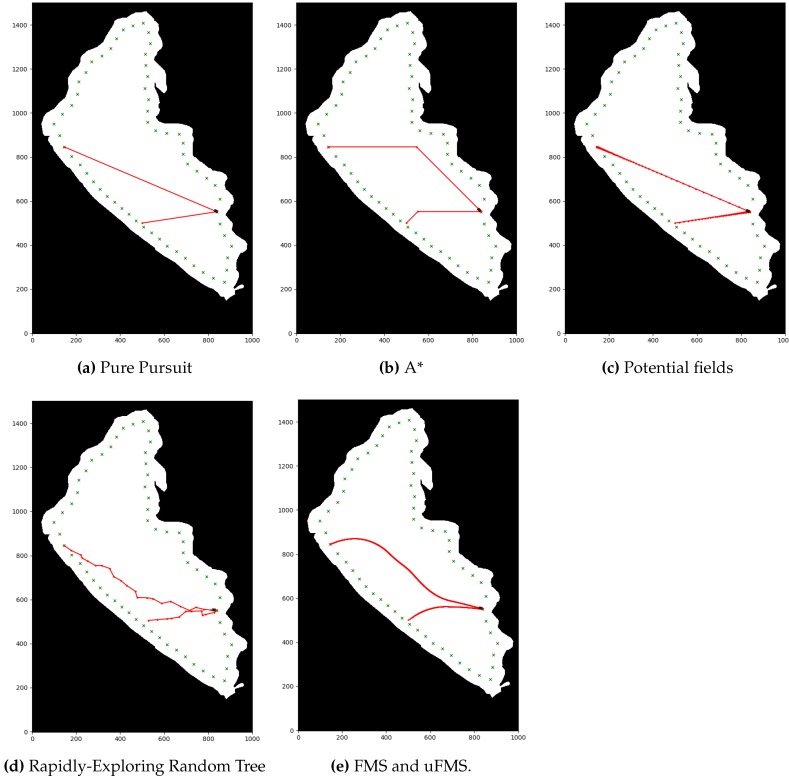
Visualization of generated paths for each of the studied methods.

First, in order to illustrate the obtained results with the different local path planning techniques are presented, the Figure 11 is presented. Figure 11a was generated example by running the PP algorithm, which are basically the routes generated by connecting two consecutive waypoints with a straight line. Second, Figure 11b was generated by running the A* algorithm, with the planned path as the red lines and the waypoints as red circles. It is important to notice that new intermediate waypoints were created with this kind of procedure, because of the nature of grid based searching algorithms, the movements of the vehicle were constrained to eight possible movements, one on each direction of the direct neighbors of the node. In this case, there was a neighbor every 45${}^{\circ }$, starting from zero, since the map was divided into regular square shapes. Figure 11c shows the resulting path using PF. As there were no obstacles in this preliminary maps, the path also had straight lines between waypoints. Then, Figure 11d shows the generated path by RRT*. It is very clear that this algorithm added many unnecessary nodes, but provided a more direct route than A* for every target. Finally, Figure 11e, shows a curved path generated by the FMS and uFMS algorithm. This curve was generated due to the smoothing effect of the Cost Function.

**Figure 12:**
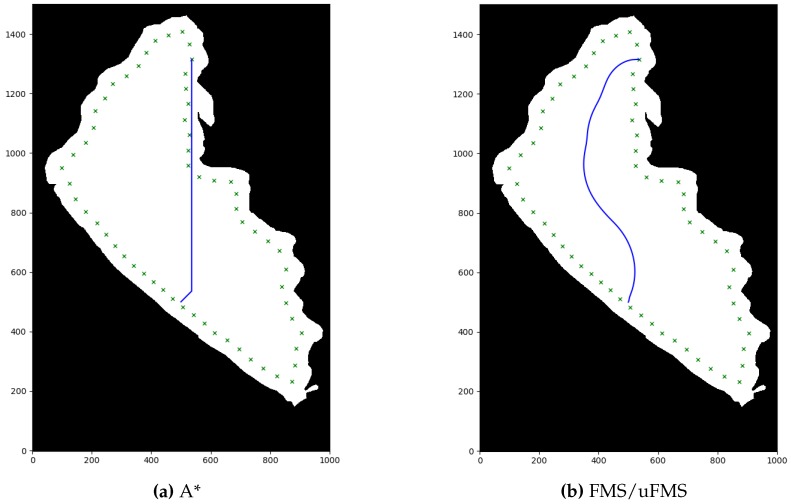
Comparison visualization of A* and FMS/uFMS planned paths

Second, the numerical results of the performance evaluation for each path planning algorithm are shown in Table 3. Although the Pure Pursuit algorithm was not a local path planner, it is included in the Table 3 as a baseline for the sake of comparison. Among the local path planning methods, RRT*, and A* took the lower average time of path planning necessary to produce outputs whenever a new obstacle was detected. The total simulation time of these path planning methods were also fairly similar. PF calculated the most direct route (lowest difference in distance), however its computation time was much higher than A* and RRT*. FMS and uFMS produced routes within the same levels of difference in distance of RRT*, both higher than A*. This statement seems to contradict the qualitative analysis that can be made by observing Figure 11b,e, but generally the uFMS planned paths were longer than the paths generated by A* method. Figure 12 shows another example of routes generated with A* and FMS/uFMS, where it is clearly seen that the planned path with A* was shorter in length but FMS/uFMS produced a safer one. The extremely high computation time for FMS makes it unsuitable to act as path planner in our scenario. However, the proposed uFMS reduced dramatically the computation time of the original FMS (see Table 3).

In terms of security level FMS and uFMS were the only methods that accomplish Level 5 of security, A* and PF methods heavily depended on the environment, they generally avoided obstacles but the ASV traveled very close to them (see Figure 12). The paths that RRT* generated contained many intermediate waypoints that were not in the same line, that caused the vehicle to take many turns and maneuvers to reach the waypoint.

**Figure 13:**
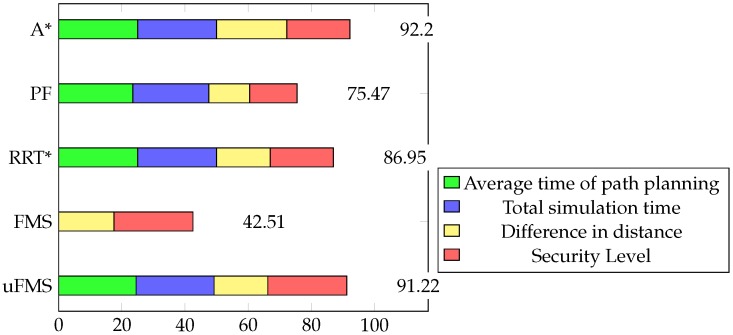
Performance metrics of the path planning techniques.

**Figure 14:**
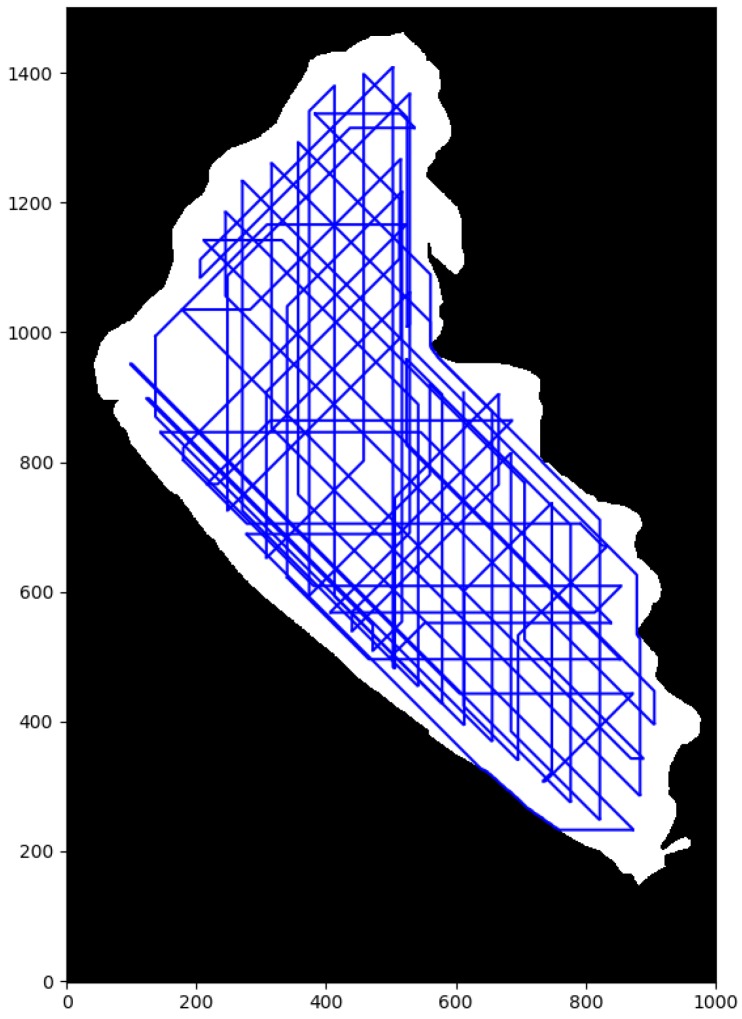
A* complete solution.

**Figure 15:**
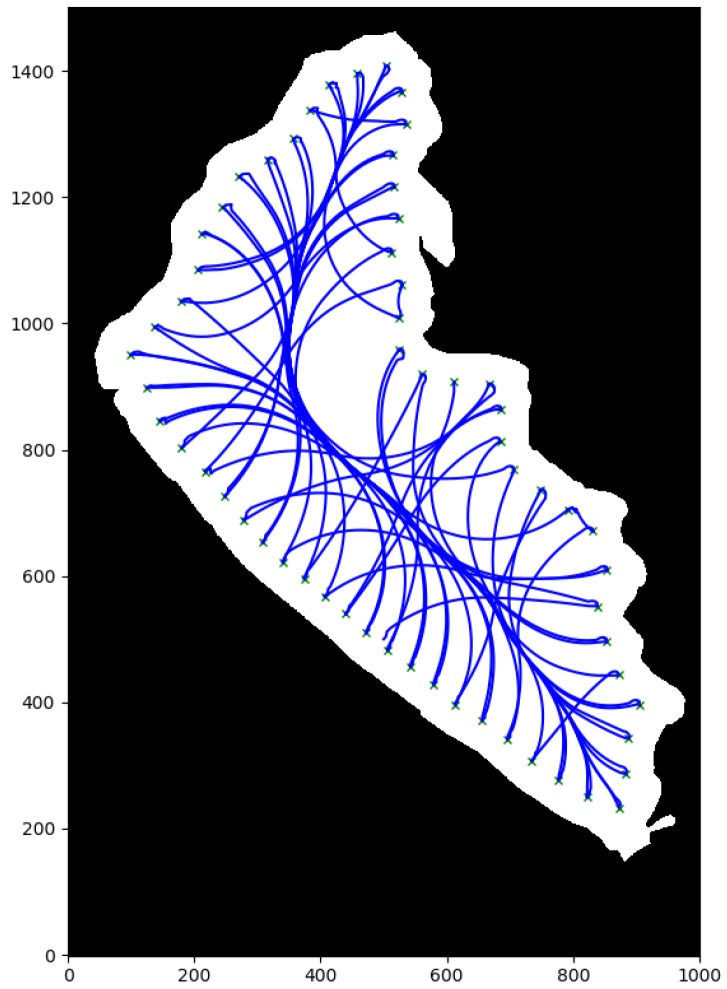
uFMS complete solution.

For a further performance analysis of these methods, all the results were weighed and added on a scale of 100, with a maximum of 25 for each criterion. A summary of the statistics of the multiple simulations are shown in Table 4 and the generated stacked bar graph can be observed in Figure 13. It is seen that the highest grades were achieved by the A* and the proposed uFMS. The A* had a slightly better performance due to a lower computational calculation time since the ASV angular rotation was performed with discrete values (multiples of 45${}^{\circ }$), while in uFMS the ASV had more rotation freedom. So it is a trade-off between both solutions, if the priority is to reduce computational time, then A* should be selected. On the other hand, if a good computational time is expected and smoother routes and greater security levels are are both required, then the uFMS should be selected. A complete solution for visiting the 60 beacons for using A* and uFMS are shown in Figure 14 and Figure 15. As a summary, the A* solution provides better coverage by creating intermediate points while the uFMS provides higher level of security by travelling around the middle of the lake. On the other hand, FMS algorithm had a significantly lower performance, mainly due to the high computational time in both path planning and total simulation time, furthermore, both of these parameters return values outside the 0–25 scale, using the maximum allowed parameters shown in Table 2.

Label Promedio Simulacion Distancia Seguridad uFMS 24.5072 24.6808 17.0271 25 FMS 0 0 17.5082 25 RRT* 24.9994 24.9753 16.9764 20 PF 23.4846 24.0492 12.9392 15 A* 24.9797 24.9749 22.241 20

## 6. Conclusions

This paper simulates and compares several local path planning techniques for an ASV developing environmental monitoring tasks in a lake. Several metrics have been defined and evaluated for each technique in order to determine the best option for the selected scenario. Findings reveal that according to the obtained results, the most attractive ones are the A* and the proposed uFMS. The first one finds shorter routes in low computational time, however the proposed uFMS provides in general better levels of security. It is worth mentioning that these performances can vary in real-life scenarios, where noises such as waves, and currents can modify the executed path, these noises can be modelled as Gaussian noises or constant forces and later be added to the simulator.

As future work, it is expected to continue testing the A* and uFMS in more complex scenarios, varying the number and sizes of obstacles, as well as the inclusion of dynamic obstacles (e.g., boats), also the effect of the environmental conditions (e.g., waves, air current, etc.) on the execution of the planned paths. Additionally, regions of interest can be included in the testing scenarios, where instead of avoiding the region the ASV is attracted to pass over this area (e.g., an area that indicates the presence of pollution or algal bloom). Another potential future work is to combine the global path planning proposed in [31] considering that the local path planning techniques presented provide routes with longer distances than the ones considered in that study. It is expected to test the reviewed and proposed algorithms in real autonomous vehicles to extend the comparison with experimental results in the lake. Finally, a third research direction is to evaluate an alternative strategy to the ring of waypoints that follows the shape of the lake. One possible alternative is to consider the task of monitoring the lake as a Partially Observed Markov Decision Process (POMDP) and solving it using a Monte-Carlo Tree Search (MCTS), selecting new waypoints according to certain parameter or reward, for example, waypoints in areas that have not been visited in a long time.

## Figures and Tables

**Table 1:** Simulation Parameters.

Number of simulations	10
Number of waypoints	60
Map size (pixels)	1000 × 1500
Pixel Size (meters)	10.33
ASV linear velocity (pix/time-step)	0.033
Time-step (μseconds)	79.68

**Table 2:** Weight normalization parameters.

Weight factor - *w*	25
Path planning maximum time (s) - $t_{ppmax}$ [s]	100
Simulation maximum time (s) - $t_{stmax}$ [s]	10,000
Maximum difference distance (pixel) - $ddif_{wmax}$ [%]	50

**Table 3:** Methods comparison.

Path Planner Algorithm	Average Time of Path Planning [s]	Total Sim. Time [s]	Difference in Distance [%]	Security Level
**Pure Pursuit**	0	110.16	0	✓
A*	0.0771	140.99	5.797	✓✓✓✓
**PF**	13.5500	933.61	0.304	✓✓✓✓
RRT*	0.0036	137.48	13.795	✓✓✓
**FMS**	1957.2800	116,741.00	14.700	✓✓✓✓✓
**uFMS**	3.1700	305.65	14.690	✓✓✓✓✓

**Table 4:** Multiple Simulations Weighted Performance Comparison.

Path Planner Algorithm	Average	Median Deviation	Standard
**Pure Pursuit**	75.0632	75.0611	0.0075
A*	92.1955	92.1813	0.2068
**PF**	75.4731	77.5890	8.9286
RRT*	86.9512	87.4272	1.6804
**FMS**	42.5081	42.0638	0.8847
**uFMS**	91.2151	90.9435	0.8192
